# A Mathematical Model for Characterizing the Biomass and the Physiological/Biochemical Indicators of *Salvia miltiorrhiza* Based on Growth-Defense Tradeoff

**DOI:** 10.3389/fpls.2021.793574

**Published:** 2022-01-04

**Authors:** Ke Wang, Zhu-Yun Yan, Yuntong Ma, Bo Li, Wei Wang, Luming Qi, Hongmei Jia, Na Li, Zhun Wang

**Affiliations:** ^1^School of Big Data and Artificial Intelligence, Chengdu Technological University, Chengdu, China; ^2^School of Pharmacy, Chengdu University of Traditional Chinese Medicine, Chengdu, China; ^3^School of Rehabilitation and Health Preservation, Chengdu University of Traditional Chinese Medicine, Chengdu, China; ^4^TCM Health Industrial Technology Institute of Traditional Chinese Medicine, Chengdu, China

**Keywords:** enzyme activities, biomass, *Salvia miltiorrhiza*, mathematical model, growth-defense tradeoff

## Abstract

Carbon(C) and nitrogen(N) metabolisms are important for plant growth and defense, and enzymes play a major role in these two metabolisms. Current studies show that the enzymes of N Metabolism, C Metabolism, and defense are correlated with biomass. Then, we conducted this research under the assumption that enzymes could characterize the relationship based on growth-defense tradeoff, and some of the enzymes could be used to represent the plant growth. From the mechanism model, we picked out 18 physiological/biochemical indicators and obtained the data from 24 tissue culture seedlings of *Salvia miltiorrhiza* (*S.miltiorrhiza*) which were grafted with 11 endophytic fungi. Then, the relationship between the biomass and the physiological/biochemical indicators was investigated by using statistical analysis, such as correlation analysis, variable screening, and regression analysis. The results showed that many physiological/biochemical indicators, especially enzyme activities, were related to biomass accumulation. Through a rigorous logical reasoning process, we established a mathematical model of the biomass and 6 key physiological/biochemical indicators, including glutamine synthetase (GS), glutamate synthase (GLS), glutamate dehydrogenase (GDH), peroxidase (POD), catalase (CAT), and soluble protein from Cobb-Douglas production function. This model had high prediction accuracy, and it could simplify the measurement of biomass. During the artificial cultivation of *S.miltiorrhiza*, we can monitor the biomass accumulation by scaling the key physiological/biochemical indicators in the leaves. Interestingly, the coefficients of Lasso regression during our analysis were consistent with the mechanism of growth-defense tradeoff. Perhaps, the key physiological/biochemical indicators obtained in the statistical analysis are related to the indicators affecting biomass accumulation in practice.

## 1. Introduction

As is known, Carbon (C) and nitrogen(N) are the main elements for the growth and defense of plants. Plants synthesize propane on the one hand through photosynthesis, which leads to starch, sucrose, and soluble sugars, and on the other hand they translate inorganic N into amino acids from ammonification, which leads to soluble proteins and are used for growth and defense in a certain C:N ratio (Lea and Morot-Gaudry, 2001). At the same time, plants consume the assimilated products of C/N metabolism through defense under biotic and abiotic stresses. Therefore, plants maintain a dynamic tradeoff between growth and defense by optimizing resource allocation through C/N metabolism and defense to enable plants to survive (Li et al., 2019).

Current studies show that some key enzyme activities of C/N metabolism have an available correlation with biomass accumulation (Noor et al., 2010). The activities of defensive enzymes show a significant correlation with adaptation to environmental stresses (Haddidi et al., 2020). Thus, the activities of sucrose phosphate synthase (SPS) and sucrose synthase (SS) in leaves, as well as the content of chlorophyll, soluble sugars, and starch are often used as important indicators to characterize the status of the C metabolism (Moriguchi et al., 1992; Verma et al., 2011). In N metabolism, nitrate reductase (NR) and nitrite reductase (NiR) translate N into NH${}_{4}^{+}$, formulate glutamate and glutamine through ammonia assimilation (Liang et al., 2018; Salehin et al., 2019). Glutaminase (GLS), glutamine synthetase (GS), glutamate synthase (GOGAT), and glutamate dehydrogenase (GDH) can promote amino acid accumulation. But with a large increase in the reduction of NH${}_{4}^{+}$ from NO${}_{3}^{-}$, a sustained raising in GDH amination activity will inhibit GLS, GS, and GOGAT activities. Thus, GLS, GS, and GOGAT have a positive influence on biomass accumulation, while GDH has a bidirectional effect on biomass accumulation (Liang et al., 2018; Salehin et al., 2019; Gonzíalez-Moro et al., 2021; Wei et al., 2021). Many soluble proteins are crucial components of enzymes. They are involved in the physiological/biochemical metabolic and are the key indicators of whether plants suffered from heavy metal stress.

In the competition of biotic and abiotic stresses, the activities of enzymes, such as GS, GDH, NR, GOGAT, NiR, and GLS, as well as the soluble protein content, are considerable indicators of the N metabolic (Salehin et al., 2019; Gonzíalez-Moro et al., 2021; Wei et al., 2021). The enzymatic activities of superoxide dismutase (SOD), peroxidase (POD), catalase(CAT), proline (Pro), phenylalanine ammonialyase (PAL), and malondialdehyde (MDA) are commonly used to characterize defense responses (Jaafar et al., 2012; Farooq et al., 2020; Sarker and Oba, 2020; Zaheer et al., 2020). The increase of the enzymes activities such as SOD, POD, CAT, and Pro can significantly improve plant growth, biomass, chlorophyll content, and gas exchange properties (Sarker and Oba, 2020; Zaheer et al., 2020), while PAL and MDA inhibit plant growth by reducing the activity of antioxidant enzymes through oxidative stress (Jaafar et al., 2012; Farooq et al., 2020). Although the relationship between C/N metabolism and plant growth defense is obvious, there is still a lack of systematic research.

**Figure 1:**
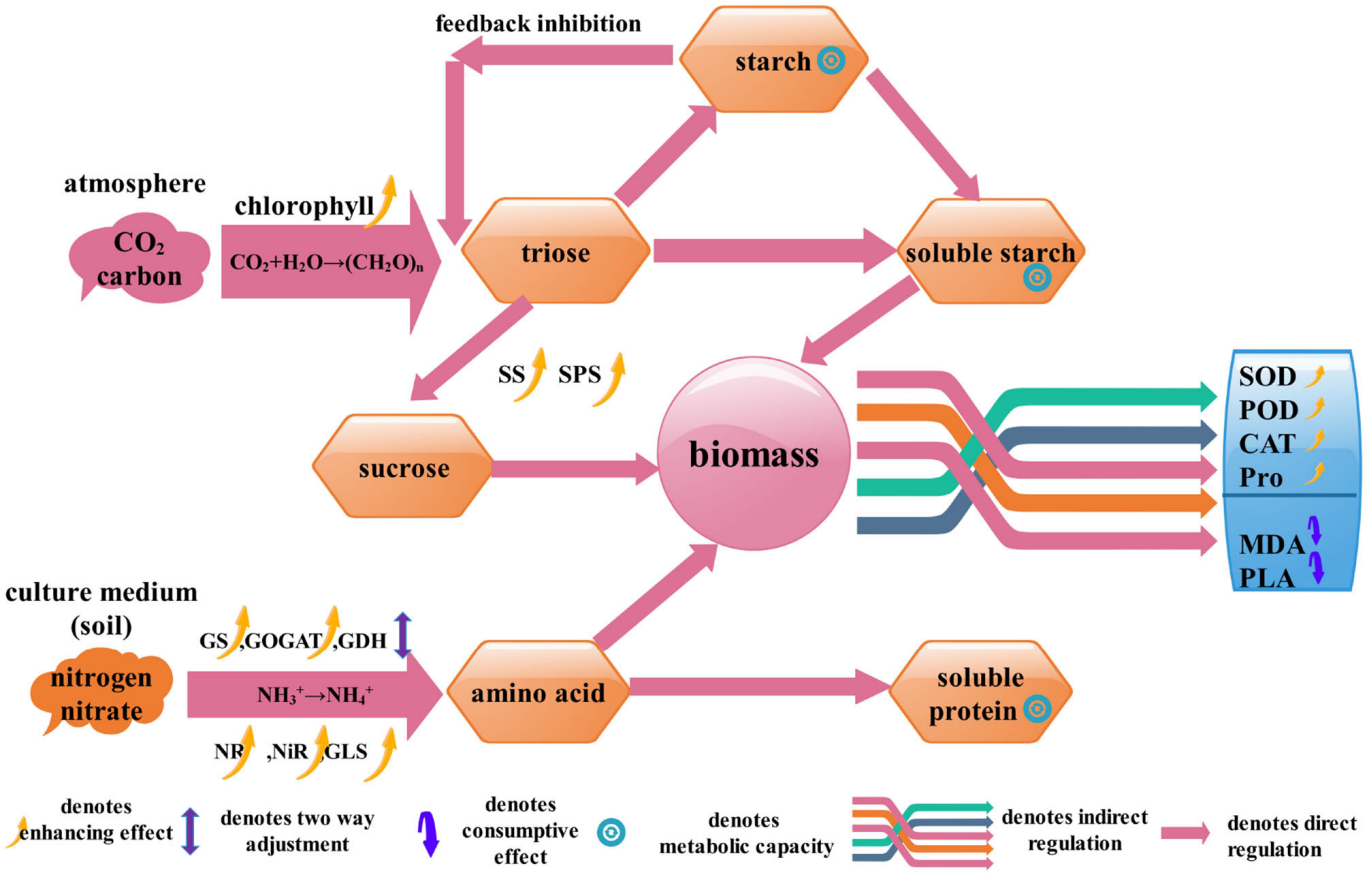
The path structure of biomass accumulation of *S.miltiorrhiza*. It shows the process of C/N metabolism and the role of the physiological/biochemical indicators in biomass accumulation.

Based on the principles of metabolism, in this study we hypothesized that the correlation among the enzymes of C/N metabolic and defense could characterize the growth-defense tradeoff, and some of the enzymes could indicate the connection between the biomass and the physiological/biochemical indicators. Then, a mechanism model was established and 18 physiological/biochemical indicators were picked out (Figure 1). Since *S.miltiorrhiza* is considerable for the treatment of coronary heart disease and cerebrovascular disease (Su et al., 2015; Ma et al., 2016; Wang et al., 2017; Li, 2018; Shi et al., 2019), this study has practical significance to guide the production of medicinal plants. We cultured 24 tissue culture seedlings of *Salvia miltiorrhiza* (*S.miltiorrhiza*) which were grafted with 11 endophytic fungi. The 11 fungi are non-pathogenic and can intervene in physiological metabolism so that we can obtain data on different growth states. Through lasso screening variable (Efron et al., 2004) and regression analysis of plant physiological and biochemical indexes and biomass, the functional relationship between *S.miltiorrhiza* biomass and physiological /biochemical indexes was found. Thus, through the monitoring of relevant indicators of *S.miltiorrhiza*, we can understand the growth status of *S.miltiorrhiza*.

## 2. Materials and Methods

### 2.1. Plant Materials

In this study, 24 tissue culture seedlings were inoculated from 11 strains of non-pathogenic endophytic fungi from 8 species of *S.miltiorrhiza* which our group obtained in previous studies (Ya-Li, 2018). The culture of tissue culture seedlings and endophytic fungal inoculation were obtained by the method in Lan et al. (2016) (daytime 25^*o*^C/nighttime 20^*o*^C, 14/10 h, light intensity 3,000 LX) where young leaves of *S.miltiorrhiza* were employed as explants. The seedlings with 3–5 roots were transplanted into artificial soil containing 50 ml of 1/2 MS medium, sealed with a sealing film, and incubated in vertical light for 3 days (Hesheng, 2000), then a small piece of fungal cake was taken with a hole punch and placed on the root of the seedlings in the culture flask, and the root of *S.miltiorrhiza* was gently punctured with a sterile needle. After 30 days of incubation, the plants were removed and the biomass and related physiological indicators were measured.

### 2.2. Determination of the Biomass

The *S.miltiorrhiza* seedlings were taken out and washed carefully to remove impurities attached to the roots. After absorbing the surface moisture with absorbent paper, weigh it and subtract the weight of seedlings when transplanting to get the net biomass.

### 2.3. Determination of the Physiological/Biochemical Indicators

The activities of SS, SPS, NR, GS, and GOGAT were determined through enzyme solution prepared from young leaves of *S.miltiorrhiza* (Deane-Drummonda et al., 1979; Islam et al., 1996; Li, 2000; Zhao et al., 2003). The contents of reducing sugar and soluble sugar were estimated by adopting 3,5-dinitrosalicylic acid method and anthrone method, respectively (Zhang, 1990; Li, 2000; Gao, 2006). The soluble protein content was assessed with the coomassie brilliant blue method (Elsharkawy et al., 2012). Chlorophyll content was determined by spectrophotometry (Strain and Svec, 1966). The activities of SOD, POD, CAT, PAL, and MDA and Pro in *S.miltiorrhiza* leaves were measured according to Zhu et al. (1983), Polle et al. (1994), and Gao (2006).

### 2.4. Statistical Analysis

#### 2.4.1. Correlation Analysis

Because of the large difference in unit and quantity, the physiological/biochemical indicators were standardized by the following function:


(1) $$\begin{array}{r} x_{ij}^{\prime }=\frac{x_{ij}-{\bar{x}}_{j}}{s_{j}}, \end{array}$$

where *x*_*ij*_ was the value of the *j*th physiological/biochemical indicator of the *i*th plant of *S.miltiorrhiza*, ${\bar{x}}_{j}$ denoted the average of *j*th indicator, and *s*_*j*_ denoted the SD of the *j*th indicator.

The correlation analysis between the physiological/biochemical indicators of *S.miltiorrhiza* and the biomass included linear correlation analysis and nonlinear correlation analysis, so the correlation coefficient matrix analysis was chosen to be used. It included three aspects: (1) analysis of the variation between the biomass and the physiological/biochemical indicators; (2) linear correlation between the biomass and the physiological/biochemical indicators; and (3) analysis of the nonlinear relationship between the biomass and the physiological/biochemical indicators.

#### 2.4.2. Systematic Analysis Based on Lasso Algorithm

According to the mechanism analysis, we tried to find the physiological/biochemical indicators related to the biomass accumulation of *S.miltiorrhiza*, so there would be duplication or the introduction of irrelevant factors. However, there are multiple covariates among various physiological/biochemical indicators, in which case least squares and partial least squares work poorly. In order to select a concise set of physiological/biochemical indicators to effectively predict biomass and improve the prediction accuracy of the model (Efron et al., 2004), Lasso regression was introduced for variable screening. The regression function was as follow:


(2) $$\begin{array}{r} y_{i}=\overset{p}{\underset{j=1}{\sum }}\beta_{j}x_{ij}^{\prime }+\varepsilon_{i},\left(i=1,2,...,n\right), \end{array}$$

where $x_{ij}^{\prime }$ denoted the *j*th physiological/biochemical indicator of the *i*th sample, *y*_*i*_ denoted the biomass of *S.miltiorrhiza* of the *i*th sample, and *p* denoted the number of physiological/biochemical indicators screened.

Because $X_{i}={\left(x_{i1},\ldots ,x_{ip}\right)}^{T},\left(i=1,2,...,p\right)$ were multicollinearity, the Lasso method was used to filter variables. The Lasso constructed a penalty function to obtain a more refined model, which made it compress some regression coefficients. Here, *l*_1_-penalty function was used for regularization estimation parameter as in Efron et al. (2004), defined as


(3) $$\begin{array}{r} \hat{\beta }\left(\lambda \right)=\text{argmin}\frac{1}{N}||Y-X\beta |{|}^{2}+\lambda \underset{j}{\sum }|\beta_{j}|, \end{array}$$

where *N* denoted the number of samples, $Y={\left(y_{1},y_{2},...,y_{N}\right)}^{T}$ denoted the biomass, $X={\left(X_{1},X_{2},...,X_{N}\right)}^{T}$ denoted the physiological/biochemical indicators, β denoted the regression coefficient, and λ≥0 denoted the penalty parameter.

#### 2.4.3. Nonlinear Regression Analysis Based on Cobb-Douglas Production Function

Based on the characteristics of important variables screened by Lasso, nonlinear regression analysis was utilized since correlations and interactions between variables still existed. Assuming that *y* denotes biomass, *A*_*i*_(*i* = 1, 2, ⋯*n*) denotes factors with positive influence coefficients, and β_*j*_(*j* = 1, 2, ⋯*m*) denotes factors with negative influence coefficients, similar to the Cobb-Douglas production model in Carter (2012), the models for the biomass and the physiological/biochemical indicators were established as follows:


(4) $$\begin{array}{r} \begin{array}{c} Y=H\cdot \frac{A_{1}^{a_{1}}A_{2}^{a_{2}}\cdots A_{n}^{a_{n}}}{B_{1}^{b_{1}}B_{2}^{b_{2}}\cdots B_{n}^{b_{m}}}, \end{array} \end{array}$$

Where *H*, *a*_1_, *a*_2_, ⋯ , *a*_*n*_, *b*_1_, *b*_2_, ⋯ , *b*_*m*_ denoted parameters to be determined by the Equation (4). Then took common logarithms on both sides of the Equation (4) at the same time, that was


(5) $$\begin{array}{l} \ln Y=\ln H+a_{1}\ln A_{1}+a_{2}\ln A_{2}+\cdots +a_{n}\ln A_{n}-b_{1}\ln B_{1}\;\\ \;-b_{2}\ln B_{2}-\cdots -b_{m}\ln B_{m}. \end{array}$$

The Equation (4) was transformed into a linear regression, from which the initial values of the nonlinear regression parameters could be obtained from the Equation (5). Then, the solution of Equation (4) could be optimized by performing a nonlinear regression.

#### 2.4.4. Data Analysis Environment

All the data were analyzed in R4.0.3 + Rstudio (Chang, 2013; Lantz, 2013). glmnet() was used for filtering variables in Lasso, ls()/nls() was used for linear/nonlinear regression analysis, and ggplot() was used for graph plotting.

## 3. Results

### 3.1. Experimental Results and Data

To analyze the relationship between the biomass and the physiological/biochemical indicators, 24 samples of *S.miltiorrhiza* were collected according to the methods described in 2.1~2.3, and the data of the 18 physiological/biochemical indicators such as chlorophyll, SS, SPS, soluble sugar, and the values of corresponding biomass were displayed in Table 1.

**Table 1:** Biomass and physiological/biochemical indicators of *S. miltiorrhiza*.

**No**	**Biomass**	**Chlorophyll**	**SS**	**SPS**	**Soluble sugar**	**Starch**	**GOGAT**	**GS**	**NR**	**NIR**	**GLS**	**GDH**	**Soluble**	**POD**	**SOD**	**CAT**	**PRO**	**PAL**	**MDA**
1	1.04	187.38	100.24	94.18	173.60	231.12	48.89	149.78	26.73	62.88	141.30	46.08	10.79	102.40	21.51	553.86	82.61	100.92	227.21
2	1.41	152.56	58.98	110.35	82.81	227.04	56.84	72.18	20.70	83.66	70.04	34.29	7.37	103.87	16.21	403.92	45.96	93.84	238.91
3	1.39	202.88	47.25	120.05	63.48	192.40	296.44	258.31	18.37	44.65	86.70	73.73	13.05	79.33	18.25	504.90	60.10	169.62	263.68
4	0.98	160.17	82.81	102.36	158.63	196.75	79.82	112.13	39.66	62.61	72.69	66.87	13.42	203.47	26.87	621.18	91.93	152.38	204.85
5	2.03	174.68	88.90	118.91	132.40	289.19	70.97	152.65	28.93	80.51	81.09	100.09	7.45	128.40	19.29	605.88	67.29	203.37	256.28
6	1.32	214.34	40.92	139.27	148.14	249.62	102.82	71.57	37.03	85.81	102.21	61.51	8.31	110.80	19.83	563.04	88.23	103.19	235.12
7	2.25	160.84	78.52	148.21	86.62	221.64	91.91	178.59	19.16	58.31	110.54	36.22	10.51	88.80	20.83	615.06	40.77	134.70	176.13
8	1.19	166.41	100.29	152.40	161.93	182.49	105.90	71.67	34.65	68.46	121.30	26.58	10.68	98.67	25.88	553.86	61.72	172.85	257.14
9	1.23	203.04	170.71	130.52	66.56	239.29	64.09	50.11	16.64	88.85	112.71	16.93	8.62	96.67	17.38	584.46	43.71	131.07	184.38
10	1.07	214.27	33.39	145.55	140.28	233.01	43.08	58.01	35.07	65.61	144.62	38.15	11.88	127.87	24.34	434.52	68.36	208.06	240.46
11	0.94	191.76	88.23	87.52	116.16	238.10	29.62	48.53	29.04	90.13	140.57	13.93	8.29	114.40	16.33	511.02	63.05	135.70	232.54
12	1.25	160.38	22.77	94.75	57.85	198.83	35.11	39.05	14.46	62.62	101.60	24.65	12.20	96.40	26.20	593.64	64.38	121.54	310.63
13	0.48	116.58	90.26	355.59	162.98	247.04	125.73	101.31	20.37	90.47	194.85	20.58	8.64	248.11	18.44	241.74	53.38	105.26	221.54
14	1.06	103.47	120.38	770.74	203.46	333.85	201.82	82.14	25.43	105.50	215.34	42.65	8.00	246.11	25.22	91.80	52.60	124.82	248.37
15	0.86	130.59	155.42	614.54	128.79	257.06	212.45	78.38	16.10	87.80	253.22	75.66	8.84	252.37	19.20	192.78	67.72	157.39	328.52
16	0.54	89.78	88.48	882.80	98.80	249.39	39.91	66.06	12.35	89.51	173.20	19.29	8.84	169.71	23.68	250.92	36.53	153.42	181.46
17	0.65	110.24	93.60	977.17	82.86	237.87	35.75	60.58	10.36	99.08	174.48	52.08	7.73	168.64	22.43	293.76	34.67	145.00	301.86
18	0.65	132.35	80.32	243.15	96.08	277.75	83.20	52.02	12.01	89.12	300.71	44.37	11.91	163.84	25.88	250.92	47.79	225.36	312.70
19	0.86	98.60	80.11	502.85	72.80	261.91	83.31	128.01	9.10	89.34	208.74	28.08	11.85	167.17	20.42	302.94	57.02	94.13	202.96
20	0.77	92.34	131.02	203.58	134.67	275.86	64.34	117.74	16.83	93.30	148.66	29.58	9.39	166.24	20.02	149.94	61.75	117.86	193.50
21	0.66	127.12	101.46	248.29	74.47	268.69	72.29	70.85	9.31	70.70	80.47	31.29	10.11	164.91	18.39	272.34	43.36	168.17	347.96
22	0.98	152.86	135.44	567.16	151.44	305.34	270.98	109.18	18.93	90.69	145.72	95.38	9.61	170.24	36.83	122.40	77.40	140.63	236.84
23	1.19	140.67	160.92	305.18	212.24	301.72	306.41	163.26	26.53	93.62	151.27	65.37	7.85	166.51	37.67	198.90	64.15	110.90	170.11
24	0.81	82.39	94.16	192.73	90.83	237.21	51.44	62.29	11.35	101.33	105.87	29.36	7.73	167.04	22.45	281.52	49.63	133.27	408.67

*In this table biomass, chlorophyll, SS, SPS, soluble sugar, starch, glutamate synthase (GOGAT), glutamine synthetase (GS), nitrate reductase (NR), nitrite reductase (NIR), glutaminase (GLS), glutamate dehydrogenase, soluble, peroxidases (POD), superoxide dismutase (SOD), catalase (CAT), proline (PRO), phenylalanine ammonialyase (PAL), malondialdehyde (MDA) are separately measured as (g), (mg· g^−1^ · FW), (mol· min^−1^ · g^−1^ · FW), (mol· min^−1^ · g^−1^ · FW), (U· g^−1^ · FW), (g· g^−1^ · FW), (U· g^−1^ · FW), (nmol· min^−1^ · g^−1^ · FW), (mol· h^−1^ · g^−1^ · FW), (mol· min^−1^ · g^−1^ · FW), (mol· h^−1^ · g^−1^ · FW), (nmol· min^−1^ · g^−1^ · FW), protein(g· ml^−1^), (U· g^−1^ · h· FW), (U· g^−1^ · h· FW), (nmol· min^−1^ · g^−1^ · FW), (U· g^−1^ · FW), (U· mg^−1^ · h), and (nmol· g^−1^ · FW), respectively*.

### 3.2. Descriptive Statistical Analysis of the Biomass and the Physiological/Biochemical Indicators

We defined biomass as the dependent variable(*y*) and chlorophyll(*x*_1_), SS(*x*_2_), SPS(*x*_3_), soluble sugar(*x*_4_), starch(*x*_5_), GOGAT(*x*_6_), GS(*x*_7_), NR(*x*_8_), NiR(*x*_9_), GLS(*x*_10_), GDH(*x*_11_), soluble protein(*x*_12_), POD(*x*_13_), SOD(*x*_14_), CAT(*x*_15_), Pro(*x*_16_), PAL(*x*_17_), and MDA(*x*_18_) as independent variables. To eliminate the difference in magnitude between variables, the criteria were standardized by using Equation (1) and then other corresponding analyses were performed.

#### 3.2.1. Differences in Some Physiological/Biochemical Indicators and Lack of Consensus in the Biomass Accumulation

**Figure 2:**
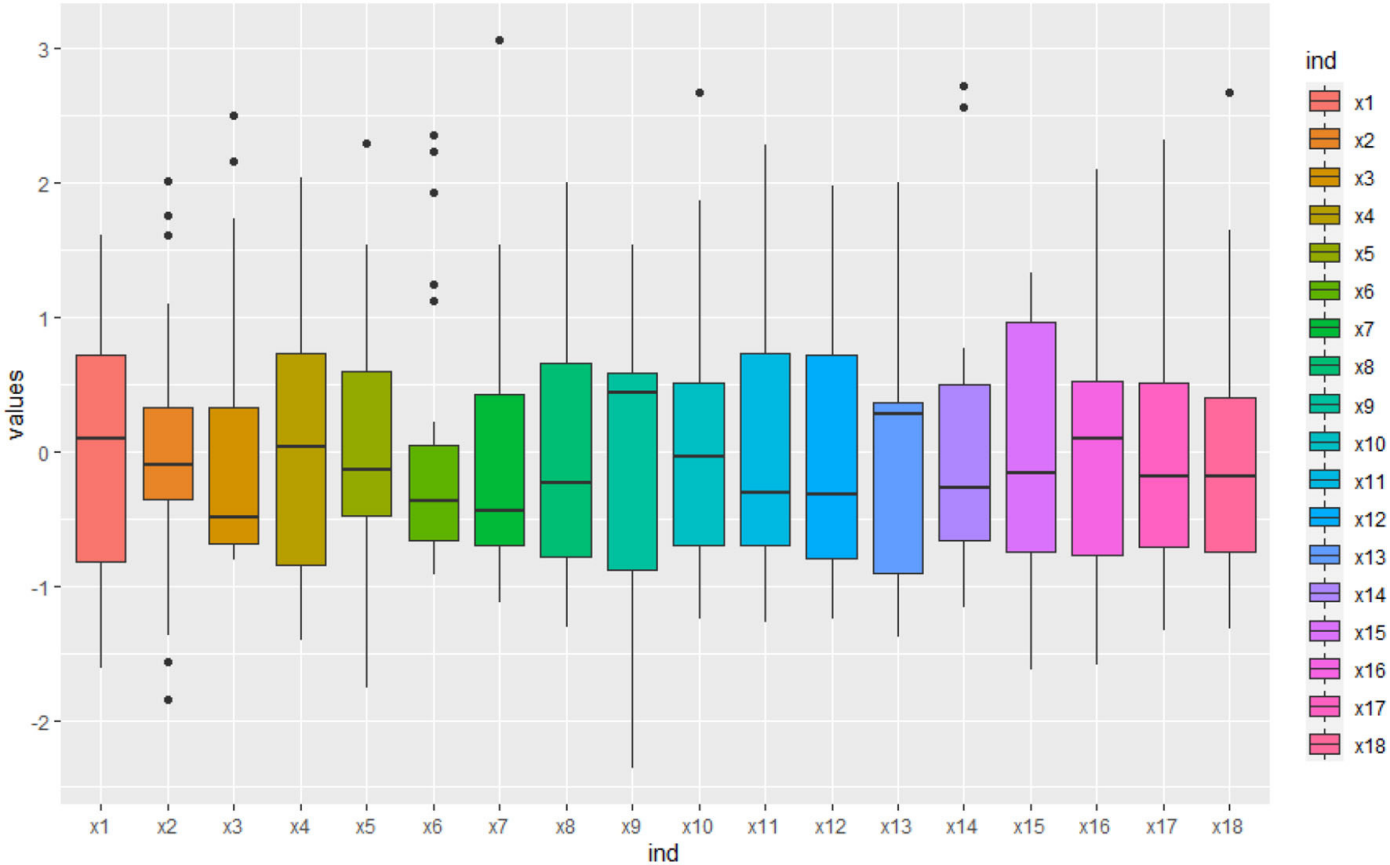
The box line diagram of the physiological/biochemical indicators. The blue curve is the regression curve, the shaded part indicates the confidence interval, and the larger the shaded area the worse the regression effect of the curve. It shows that some physiological/biochemical indicators lack consensus in the biomass accumulation of *S.miltiorrhiza*.

Some physiological/biochemical indicators were influenced by individual plants, especially SS(*x*_2_) and GOGAT(*x*_6_) were very different (Figure 2). It indicated that they lacked consensus in the biomass accumulation of *S.miltiorrhiza* and there was uncertainty among indicators.

#### 3.2.2. Many Physiological/Biochemical Indicators Have a Linear (or Nonlinear) Correlation With the Biomass

**Figure 3:**
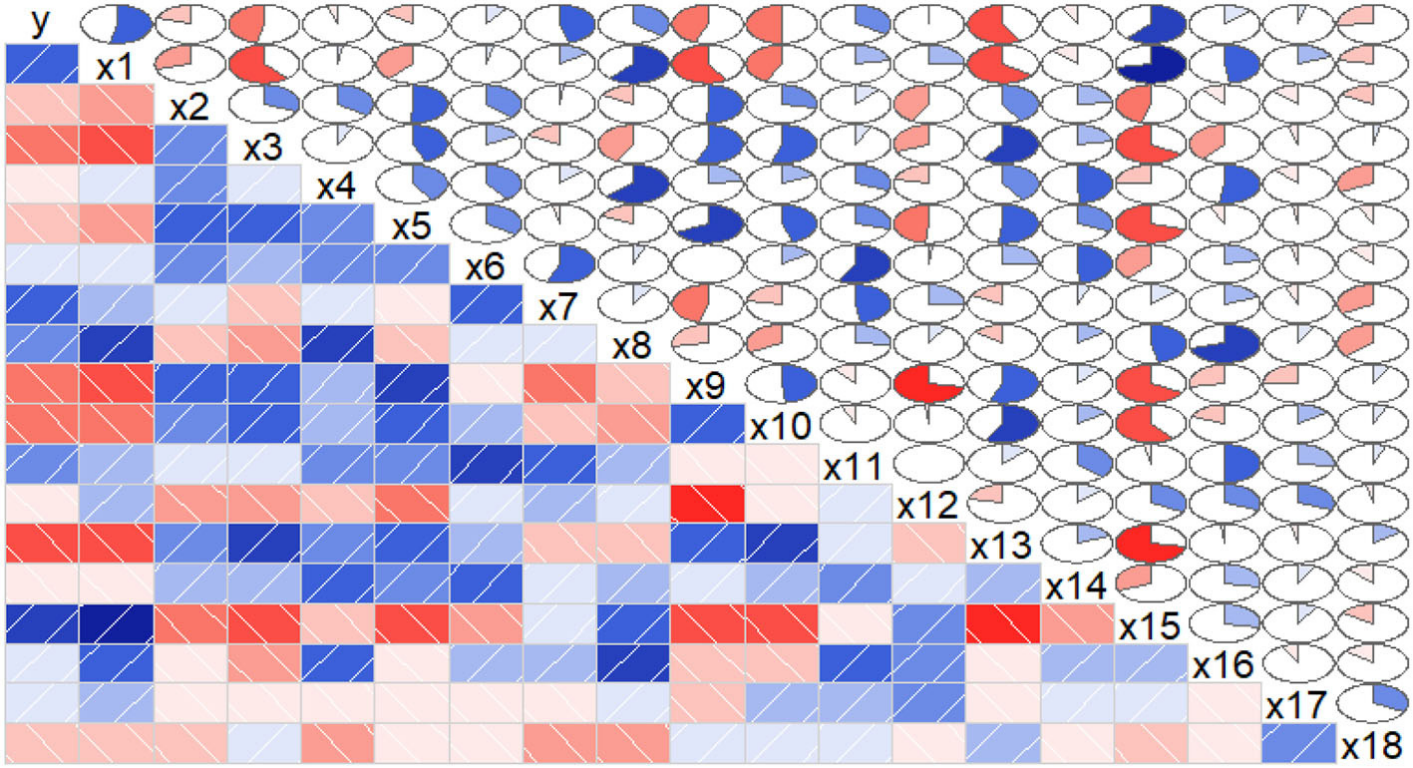
Correlations between the biomass and the physiological/biochemical indicators of *S.miltiorrhiza* biomass. The blue rectangles represent a positive correlation, the red rectangles represent a negative correlation. The area of the circle represents the degree of correlation. According to correlograms shown in this figure, there were correlations among the biomass and the physiological/biochemical indicators.

From the result of correlation analysis, we can see there was a positive correlation of the biomass(*y*) with chlorophyll(*x*_1_) and CAT(*x*_15_) (Figure 3), and a negative correlation with GLS(*x*_10_) and POD(*x*_13_). Considering the relationship between the physiological/biochemical indicators, chlorophyll(*x*_1_), NR(*x*_8_), and CAT(*x*_15_) were positively correlated, while chlorophyll(*x*_1_) was negatively correlated with SPS(*x*_3_), NiR(*x*_9_), and POD(*x*_13_). SPS(*x*_3_) was positively correlated with CAT(*x*_15_), while SPS(*x*_3_) was negatively correlated with NiR(*x*_9_), GLS(*x*_10_) and POD(*x*_13_). Similarly, these results indicated that linear correlations existed between the biomass and the physiological/biochemical indicators.

**Figure 4:**
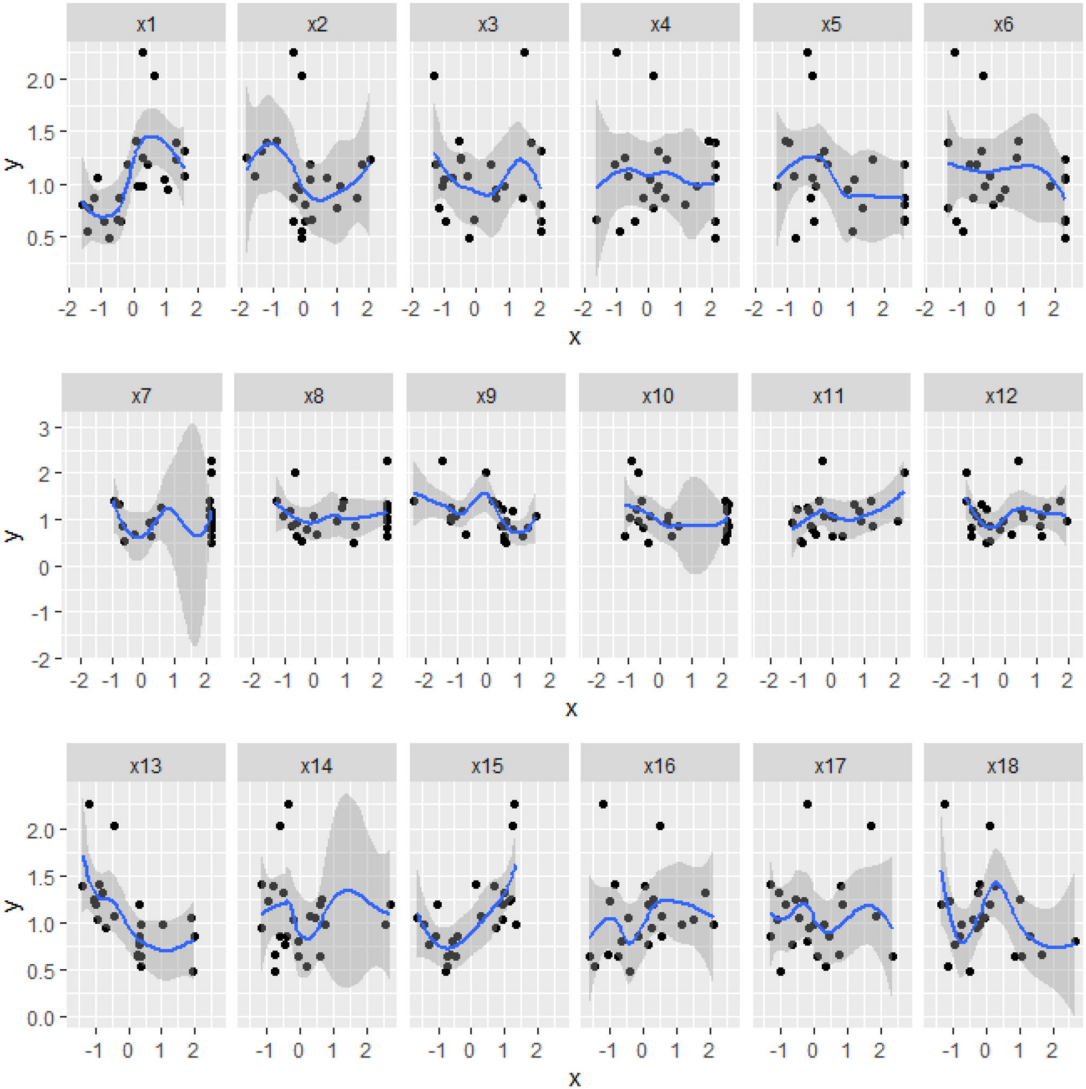
The scatter and regression curves of the biomass and the physiological/biochemical indicators of *S.miltiorrhiza*. The horizontal axis of *x*_*i*_, is the physiological/biochemical indicator of *i* and the vertical axis is *y*, which is the biomass of *S.miltiorrhiza*. The blue curve is the regression curve, the shaded part indicates the confidence interval, and the larger the shaded area indicates the worse regression effect of the curve.

There was a certain nonlinear relationship (Figure 4) between the biomass and the physiological/biochemical indicators. For instance, there was a nonlinear relationship between the biomass(*y*), and NiR(*x*_9_), and soluble protein(*x*_12_). Thereby, biomass could not be expressed by a single enzyme activity or physiological/biochemical indicator, but by a combination of some effective physiological/biochemical indicators (Figures 3, 4). Therefore, it was necessary to systematically analyze the relationship between the physiological/biochemical indicators and the biomass.

### 3.3. Some Physiological/Biochemical Indicators Play a Major Role in the Biomass Accumulation

**Figure 5:**
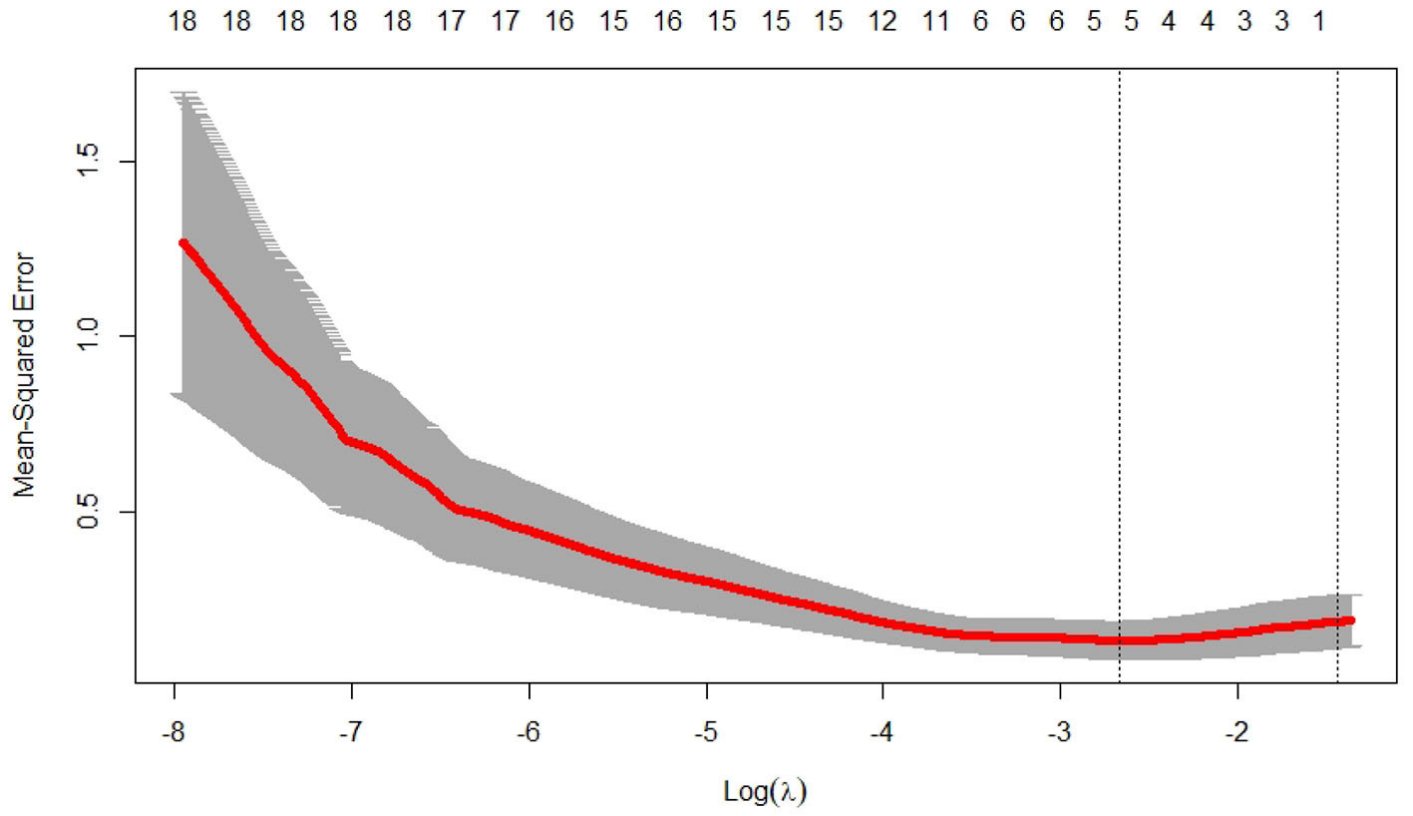
Lasso regression analysis number of iterations vs. mean square error. It shows that the optimal λ value is obtained in 5th step.

We used the glmnet() of the R software to calculate the Lasso model (parameters set to default values) and optimized the model and function by cv.glmnet() to obtain the results shown in Figure 5 and Table 2.

**Table 2:** Nonzero regression coefficient of Lasso.

***x*_7_**	***x*_10_**	***x*_11_**	***x*_12_**	***x*_13_**	***x*_15_**
1.385218e-03	–1.205714e-05	1.999661e-03	–4.228444e-03	–1.422853e-03	7.330554e-04

The results showed that GS(*x*_7_), GDH(*x*_11_), and CAT(*x*_15_) were positively correlated with biomass and acted as promoters when the whole system was considered. GLS(*x*_10_), POD(*x*_15_), and soluble protein(*x*_13_) were negatively correlated with biomass accumulation and acted as inhibitors.

### 3.4. Some Key Physiological/Biochemical Indicators Are Able to Express the Biomass Well

A nonlinear regression analysis was performed by using the Douglas production model with the influencing factors of GS(*x*_15_), GLS(*x*_15_), GDH(*x*_15_), soluble protein(*x*_15_), POD(*x*_15_), and CAT(*x*_15_), and biomass. Took 5/6 of the data as the training set and 1/6 as the verification set, repeated the training 1,000 times, and selected the regression equation with the smallest verification set error. The model was as follows:


(6) $$\begin{array}{r} Y=5.05853\times \frac{x_{7}^{0.24737}x_{11}^{0.09026}x_{15}^{0.22631}}{x_{10}^{0.14776}x_{12}^{0.64021}x_{13}^{0.44381}}. \end{array}$$

The predicted values of biomass could be obtained from Equation (6) and the results were shown in Table 2. The test showed that the prediction effect of the model was good (Tables 3, 4). It showed that the regression equation was relatively successful and was able to predict the corresponding the biomass from the 6 important physiological/biochemical indicators.

**Table 3:** The results of the training set of the biomass and the key physiological/biochemical indicators.

**x7**	**x10**	**x11**	**x12**	**x13**	**x15**	**y**	**hat_y**	**absolute_error**
48.53	140.57	13.93	8.29	114.4	511.02	0.94	1.043	0.103
62.29	105.87	29.36	7.73	167.04	281.52	0.81	0.956	0.146
71.67	121.3	26.58	10.68	98.67	553.86	1.19	1.151	−0.039
82.14	215.34	42.65	8	246.11	91.8	1.06	0.609	−0.451
112.13	72.69	66.87	13.42	203.47	621.18	0.98	0.969	−0.011
258.31	86.7	73.73	13.05	79.33	504.9	1.39	1.728	0.338
101.31	194.85	20.58	8.64	248.11	241.74	0.48	0.72	0.24
117.74	148.66	29.58	9.39	166.24	149.94	0.77	0.817	0.047
71.57	102.21	61.51	8.31	110.8	563.04	1.32	1.425	0.105
178.59	110.54	36.22	10.51	88.8	615.06	2.25	1.63	−0.62
50.11	112.71	16.93	8.62	96.67	584.46	1.23	1.198	−0.032
152.65	81.09	100.09	7.45	128.4	605.88	2.03	1.898	−0.132
60.58	174.48	52.08	7.73	168.64	293.76	0.65	0.934	0.284
128.01	208.74	28.08	11.85	167.17	302.94	0.86	0.796	−0.064
72.18	70.04	34.29	7.37	103.87	403.92	1.41	1.476	0.066
66.06	173.2	19.29	8.84	169.71	250.92	0.54	0.771	0.231
39.05	101.6	24.65	12.2	96.4	593.64	1.25	0.952	−0.298
109.18	145.72	95.38	9.61	170.24	122.4	0.98	0.832	−0.148
149.78	141.3	46.08	10.79	102.4	553.86	1.04	1.387	0.347
78.38	253.22	75.66	8.84	252.37	192.78	0.86	0.68	−0.18

**Table 4:** The results of the validation set of the biomass and the key physiological/biochemical indicators.

**x7**	**x10**	**x11**	**x12**	**x13**	**x15**	**y**	**hat_y**	**absolute_error**
58.01	144.62	38.15	11.88	127.87	434.52	1.07	0.866	−0.204
52.02	300.71	44.37	11.91	163.84	250.92	0.65	0.606	−0.044
70.85	80.47	31.29	10.11	164.91	272.34	0.66	0.869	0.209
163.26	151.27	65.37	7.85	166.51	198.9	1.19	1.134	−0.056

## 4. Discussion

Carbon/nitrogen metabolism are critical in growth and defense (Li et al., 2019). Adequate management of the plant in C/N metabolism and growth defense have a significant impact on crop productivity. In this study, we constructed a growth mechanism model by *S.miltiorrhiza* and picked out the relationship between the 18 physiological/biochemical indicators and the C/N metabolism, with the aim of finding important metabolic traits as an indication of the biomass (Table 2).

The variability of the physiological/biochemical indicators was analyzed by box-line plots (Figure 2), and it was observed that some indicators showed no consensus. That was, some physiological/biochemical indicators were greatly affected by individuals. In particular, SS(*x*_2_) and GOGAT(*x*_6_) were more discrete. SS(*x*_2_) was an important enzyme catalyzing sucrose synthesis in plants, and sucrose was broken down into soluble sugars for drought resistance in plants on the one hand, and synthesized biomass with soluble proteins on the other hand, making SS showed uncertainty on the biomass (Kaur et al., 2006; Zhang et al., 2018). Similarly, GOGAT(*x*_6_) consuming amino acids used by plants for the biomass accumulation during growth defense, formed aspartic acid, which was used for plant drought resistance under the action of Pro and producing glutamate, soluble protein, which leads to biomass accumulation. The uncertainty of the two conversion pathways, SS(*x*_2_), and GOGAT(*x*_6_), prevents them from being used as important indicators for characterizing the biomass (Wei et al., 2021).

There were linear(or nonlinear) correlations between the biomass and the physiological/biochemical indicators (Figures 2, 3), indicating interdependence in the growth-defense tradeoff of plants. For example, there was a positive correlation between biomass(*y*) and chlorophyll(*x*_1_)/CAT(*x*_15_), indicating that chlorophyll was the main substance for photosynthesis in plants, and it played an important role in C metabolism (Luo, 2018). Although CAT(*x*_15_) was mainly used in the defense, it reduced the toxicity of H_2_O_2_ (Abogadallah, 2010; Sarker and Oba, 2020), and maintained normal growth and defense. The biomass was negatively correlated with some physiological/biochemical indicators, such as GLS(*x*_6_) promoting amino acid accumulation, and POD(*x*_13_) depleting C and N assimilation products, affecting the biomass accumulation. POD(*x*_13_) reduces H_2_O_2_ and converts carbohydrates to lignin, improving the physical defense barrier and depleting assimilates required for growth. Considering the relationship between the physiological/biochemical indicators, chlorophyll(*x*_1_), NR(*x*_15_), and CAT(*x*_15_) were positively correlated, while chlorophyll(*x*_15_) was negatively correlated with SPS(*x*_3_), NiR(*x*_9_), and POD(*x*_13_) (Figure 3). On the one hand, chlorophyll is used by plants for photosynthesis to convert CO_2_ in the air into C required by plants, and on the other hand, about 90% of N in plants, coming from biological N fixation, is reduced from nitrate to nitrite in soil by NR. Plant cells can rapidly transfer nitrite produced by nitrate reduction from the stroma to the chloroplasts of leaf cells or the plastids of root cells and are reduced in the chloroplasts by NiR to ammonia, while the potential toxicity of nitrite is broken down by CAT(*x*_15_). Chlorophyll(*x*_1_), NR(*x*_8_), NiR(*x*_9_), and CAT(*x*_15_) collaborate with each other in C/N metabolism as well as in the growth-defenses tradeoff (Zhou et al., 2021). SPS(*x*_3_) and SS(*x*_2_) are both important enzymes for catalyzing sucrose synthesis in plants, however, SPS exhibiting different characteristics from sucrose SPS SS(*x*_2_) was positively correlated with CAT(*x*_15_), indicating that it promoted the biomass accumulation (Figure 3). While SPS was negatively correlated with NiR(*x*_9_), GLS(*x*_10_), and POD(*x*_13_)(Figure 3). It showed inhibition of biomass accumulation, but NiR(*x*_15_) reduced nitrate in soil and then synthesized NH${}_{4}^{+}$ for the biomass formation, and GLS(*x*_10_) formed NH${}_{4}^{+}$ into amino acids, which were against the previous analysis. All of these analyses indicated that biomass accumulation was a relatively complex process that required systematic analysis of the physiological/biochemical indicators affecting the biomass accumulation.

By Lasso analysis (Figure 5 and Table 2), GS(*x*_7_) and GDH(*x*_11_) were positively correlated with biomass (Table 3), indicating that GS(*x*_7_) and GDH(*x*_11_) were the main substrate in the seedling stage facilitate protein synthesis with amino acids and were conducive to coordinate C metabolism (Kaur et al., 2006), which was consistent with the rapid assimilation of N in early growth and laid the foundation for high rate C assimilation in later growth stages (Salehin et al., 2019; Gonzíalez-Moro et al., 2021; Wei et al., 2021). GS(*x*_7_) was responsible for primary NH${}_{4}^{+}$ assimilation as well as germination, and elevating of GS(*x*_7_) in leaves contributed to the conversion of inorganic NH${}_{4}^{+}$ to organic N (Lea and Morot-Gaudry, 2001). There was a positive correlation between GDH(*x*_11_) and total amino acid content in the root system (Gonzíalez-Moro et al., 2021). When GDH(*x*_11_) was sufficiently induced in leaves, it would indicated substrate specificity in plants with other enzymes. Although GLS facilitated amino acid accumulation, it was negatively correlated with the biomass (Table 3), possibly due to the lack of sufficient reducing agent(NADPH) or the unavailability of reducing agent(NADPH), making it possible that NH${}_{3}^{+}$ might not attack the ketone group, leading to its release into the environment (Yoneyama et al., 2015). Soluble proteins were negatively correlated with biomass (Table 3), indicating that soluble proteins would consume amino acids used for biomass accumulation. POD was negatively correlated with biomass (Table 3), indicating that glutamate synthesis, as well as the synthesis of metabolic enzymes represented by POD, consumed N and C assimilation products, thus affecting the biomass accumulation. POD reduced H_2_O_2_ and converted carbohydrates to lignin, improving the physical defense barrier and depleting assimilates required for growth. However, despite its synthesis of N and C assimilation products, CAT(*x*_15_) specifically reduced the toxicity of H_2_O_2_ (Abogadallah, 2010; Sarker and Oba, 2020), maintained cell membrane stability, and promoted growth and defense. Meanwhile, it was a positive correlation between CAT(*x*_15_) and biomass(*y*) (Table 3).

The relationship between the physiological/biochemical indicators in the biomass accumulation mechanism model was consistent with the results of the Lasso model analysis. Our analysis not only illustrated that the biomass accumulation was related to some key physiological/biochemical indicators, but also the characteristics of these enzymes were the same as those of Lasso analysis. For example, GS(*x*_7_) and GDH(*x*_10_), which had positive coefficients in the Lasso model, were also present in the routes of C/N metabolism to promote biomass accumulation, and GLS(*x*_11_) and POD(*x*_13_), which had negative coefficients in the Lasso model, were also present in the routes of defense to consume the C and N and affect the biomass accumulation (Lea and Morot-Gaudry, 2001). Furthermore, although CAT(*x*_15_) consumed C and N on the defense route, its negative synergistic effect with POD(*x*_13_), or competition for substrates, could explain this phenomenon. Thereby, perhaps increasing GS(*x*_7_), GDH(*x*_10_), and CAT(*x*_15_) activities, or decreasing GLS(*x*_11_) and POD(*x*_13_) activities, would be beneficial to promote biomass accumulation of *S.miltiorrhiza*.

Finally, we established a functional equation between the 6 key physiological/biochemical indicators and the biomass based on the Cobb-Douglas economic model. Although Cobb-Douglas economic model is an economic mathematical model used to predict the production of national and regional industrial systems or large enterprises and analyze the ways to develop production, this study skillfully uses it to establish the relationship between physiological/biochemical indicators and biomass. Interestingly, the correlation between the physiological/biochemical indicators and the biomass in this equation was the same as that obtained by Lasso regression. That is GS(*x*_7_), GDH(*x*_11_), and CAT(*x*_15_) were positively correlated with the biomass, and GLS(*x*_10_), POD(*x*_12_), and soluble protein(*x*_13_) were negatively correlated with biomass accumulation. The biomass predicted from the physiological/biochemical indicators of this equation had less error. In the artificial cultivation of *S.miltiorrhiza*, only the values of the key physiological/biochemical indicators of the above-ground parts are needed to obtain the corresponding biomass.

## 5. Conclusion

This study provides a rigorous logical reasoning process in terms of the selection of factors affecting biomass accumulation, the screening of key factors, and the establishment and validation of regression models. The method is applicable not only to metabolic engineering but also to phenomena with similar mechanistic features, such as the relationship between the activities and soil environment, and the self-organization of microbial communities. In addition, the generalized application of Lasso regression and the Cobb-Douglas production model used in this study provides a powerful tool for a comprehensive and systematic study of growth and active ingredient synthesis.

## Data Availability Statement

The original contributions presented in the study are included in the article/supplementary material, further inquiries can be directed to the corresponding author.

## Author Contributions

ZY and KW obtained funding. BL, HJ, NL, and ZW designed the experiments and analyzed the data, with the physiology support from YM and LQ generated the structure diagram of biomass accumulation mechanism, WW modified Lasso and nonlinear regression algorithm. KW completed all the program design and graphics drawing. KW and ZY wrote this study with the contributions of all the authors, who also approved the final version prior to submission.

## Funding

This study was supported by Sichuan Science and Technology Program (2021 YFS0045), Special Scientific and Technological Research Project of Sichuan Province Administration of Traditional Chinese Medicine (Project 2018QN013), State Key Laboratory of Characteristic Chinese Medicine Resources in Southwest China, School of Pharmacy, Chengdu University of Traditional Chinese Medicine, Chengdu, China.

## Conflict of Interest

The authors declare that the research was conducted in the absence of any commercial or financial relationships that could be construed as a potential conflict of interest.

## Publisher's Note

All claims expressed in this article are solely those of the authors and do not necessarily represent those of their affiliated organizations, or those of the publisher, the editors and the reviewers. Any product that may be evaluated in this article, or claim that may be made by its manufacturer, is not guaranteed or endorsed by the publisher.

## Abbreviations

C: carbon
CAT: catalase
GDH: glutamate dehydrogenase
GLS: glutaminase
GOGAT: glutamate synthase
GS: glutamine synthetase
MDA: malondialdehyde
N: nitrogen
NiR: nitrite reductase
NR: nitrate reductase
PAL: phenylalanine ammonialyase
PGA: 3- phosphoglyceric acid
POD: peroxidases
Pro: proline
NADPH: reducing agent
*S.miltiorrhiza*: *Salvia miltiorrhiza*
SOD: superoxide dismutase
SPS: synthase
SS: sucrose synthase.
